# Differentially Expressed Wound Healing-Related microRNAs in the Human Diabetic Cornea

**DOI:** 10.1371/journal.pone.0084425

**Published:** 2013-12-20

**Authors:** Vincent A. Funari, Michael Winkler, Jordan Brown, Slobodan D. Dimitrijevich, Alexander V. Ljubimov, Mehrnoosh Saghizadeh

**Affiliations:** 1 Genomics Core, Cedars-Sinai Medical Center, Los Angeles, California, United States of America; 2 University of California Los Angeles, Los Angeles, California, United States of America; 3 Eye Program, Regenerative Medicine Institute, Department of Biomedical Sciences, Cedars-Sinai Medical Center, Los Angeles, California, United States of America; 4 Department of Integrative Physiology, University of North Texas Health Science Center, Fort Worth, Texas, United States of America; University of Florida, United States of America

## Abstract

MicroRNAs are powerful gene expression regulators, but their corneal repertoire and potential changes in corneal diseases remain unknown. Our purpose was to identify miRNAs altered in the human diabetic cornea by microarray analysis, and to examine their effects on wound healing in cultured telomerase-immortalized human corneal epithelial cells (HCEC) *in vitro*. Total RNA was extracted from age-matched human autopsy normal (n=6) and diabetic (n=6) central corneas, Flash Tag end-labeled, and hybridized to Affymetrix® GeneChip® miRNA Arrays. Select miRNAs associated with diabetic cornea were validated by quantitative RT-PCR (Q-PCR) and by *in situ* hybridization (ISH) in independent samples. HCEC were transfected with human pre-miR^TM^miRNA precursors (h-miR) or their inhibitors (antagomirs) using Lipofectamine 2000. Confluent transfected cultures were scratch-wounded with P200 pipette tip. Wound closure was monitored by digital photography. Expression of signaling proteins was detected by immunostaining and Western blot. Using microarrays, 29 miRNAs were identified as differentially expressed in diabetic samples. Two miRNA candidates showing the highest fold increased in expression in the diabetic cornea were confirmed by Q-PCR and further characterized. HCEC transfection with h-miR-146a or h-miR-424 significantly retarded wound closure, but their respective antagomirs significantly enhanced wound healing vs. controls. Cells treated with h-miR-146a or h-miR-424 had decreased p-p38 and p-EGFR staining, but these increased over control levels close to the wound edge upon antagomir treatment. In conclusion, several miRNAs with increased expression in human diabetic central corneas were found. Two such miRNAs inhibited cultured corneal epithelial cell wound healing. Dysregulation of miRNA expression in human diabetic cornea may be an important mediator of abnormal wound healing.

## Introduction

Recently, a family of small noncoding RNAs, microRNAs (miRNAs), have emerged as important regulators in normal and pathological conditions. MicroRNAs play a critical role in the regulation of gene expression at the post-transcriptional level usually resulting in gene silencing via translational repression or target degradation [1-4]. Although a growing number of miRNAs have been identified, relatively little is known about their biological functions and their target mRNAs. Emerging studies indicate that miRNAs are important regulators in a variety of developmental, physiological and pathological processes including cell proliferation, migration, differentiation, apoptosis, inflammation and stem cell maintenance [5-10]. In addition, there is substantial evidence supporting the involvement of miRNAs in many diseases including cancer [11-13], cardiovascular disorders [14,15], and diabetes [16-19], which may have an impact on future treatments of such diseases. 

The critical role of miRNAs in eye development has been shown using conditional Dicer knockout mice, which failed to develop discernible lens and had poorly stratified corneal epithelium [20]. Several retina-specific miRNAs have also been identified in human and mouse by microarray analysis and by a 3′UTR target finding approach of known retinal genes [7,8]. To date, few studies have addressed the role of these regulators in the eye [6-8,16,17].

Diabetes mellitus (DM) has significant negative effects in the cornea, which can often be sight threatening. Diabetes affects all the layers of the cornea and corneal nerves [21-24]. Corneal abnormalities such as epithelial defects and fragility, recurrent epithelial erosions, decreased sensitivity, abnormal wound repair, increased susceptibility to injury and infection, ulcers, edema, and increased auto-fluorescence have been clinically observed in DM patients with or without diabetic retinopathy (DR) [23,24]. Mechanisms responsible for these changes are still not well understood, which hampers the development of effective treatments, and calls for further studies to understand the causes of corneal diabetic pathology.

To date, few studies addressed the expression and function of corneal miRNAs [25-29]. Topographical differences in expression between various ocular surface compartments (central cornea, limbus, and adjacent conjunctiva) were recently described for several corneal miRNAs [30,31]. No data are available yet on miRNA changes in common corneal diseases including diabetic keratopathy.

To fill this gap, we performed a global microarray analysis of miRNA expression in normal and diabetic human corneas and successfully identified and confirmed by quantitative real-time RT-PCR (Q-PCR) several miRNAs differentially expressed in diabetic corneas. A wound healing study of two overexpressed miRNAs in a non-transformed human corneal epithelial cell line revealed their role in regulating wound healing that is impaired in the diabetic cornea.

## Materials and Methods

### Tissues

Age-matched human autopsy normal, diabetic, and DR corneas and whole eyes were obtained from the National Disease Research Interchange (NDRI, Philadelphia, PA); donor identity was withheld by the supplier. NDRI has a human tissue collection protocol approved by a managerial committee and subject to National Institutes of Health oversight. This work was covered by an exempt IRB protocol EX-1055 from Cedars-Sinai Medical Center. Corneas received in Optisol storage medium (Chiron Vision, Claremont, CA) within 24 hours of donor death were trephined, immediately frozen in liquid nitrogen, and stored at -80°C. 

### Isolation of Total RNA

Total RNA including low molecular weight (LMW) RNA was extracted from age-matched human autopsy normal and diabetic 8.5 mm central corneal buttons using the Ambion mirVana^TM^miRNA isolation kit (Life Technologies, Carlsbad, CA) according to the manufacturer's instructions and were stored at -80°C.The RNA quality was assessed using a NanoDrop ND-1000 spectrophotometer (Thermo Scientific, West Palm Beach, FL), Agilent 2100 system (Agilent Technologies, Santa Clara, CA), and a Qubit 2.0 fluorometer (Life Technologies). 

### Microarray Probe Synthesis and Hybridization

For miRNA microarray analysis, six normal corneas (mean patient age, 79.8 ± 7.47 [mean ± SD] years) and six diabetic with or without retinopathy (mean patient age, 68.6 ± 13.19 years) corneas were used (Table 1). The profiles of large and small ribosomal subunits were used as surrogates for miRNA quality. Each of the 12 samples had an Agilent RNA integrity score (RIN) ≥ 8.5. Total RNA containing LMW RNAs was labeled with GeneChip® miRNA Arrays 1.0 containing 812 human miRNAs as well as 500 snoRNAs and scaRNAs (Affymetrix, Santa Clara, CA) according to manufacturer’s guidelines.

**Table 1:** Donor characteristics.

**Case number**	**Age**	**Gender**	**Cause of death**	**DM duration, years**
N 01-45	72	M	Congestive heart failure	N/A
N 04-03	80	M	Pneumonia	N/A
N 04-66	81	F	Myocardial infarction	N/A
N 05-33	77	F	Cardiac arrest	N/A
N 07-10	74	M	Congestive heart failure	N/A
N 04-33	59	F	Unknown	N/A
N 04-67	95	M	Multi-organ failure	
N05-05	81	M	Parkinson’s disease	N/A
N 05-32	56	F	Lung cancer	N/A
N 13-01	78	F	Heart disease	N/A
N 13-02	57	F	Hepatic encephalopathy	N/A
N 13-08	90	F	Congestive heart failure	N/A
DM 03-25	73	F	Myocardial infarction	Unknown
DM 07-06	83	M	Congestive heart failure	10
DM 07-18	65	F	Myocardial infarction	33
DR 04-57	64	F	Respiratory failure	35
DR 04-116	43	M	Subarachnoid hemorrhage	33
DR 07-15	65	F	Myocardial infarction	33
DM 07-07	66	F	Respiratory failure	12
DM 07-16	69	M	Coronary artery disease	10
DM 13-01	78	F	Pulmonary disease	10
DM 13-13	78	M	Respiratory failure	31
DM 13-22	82	M	Respiratory failure	20

N, normal; DM, diabetic mellitus; DR, diabetic retinopathy; M, male; F, female.

### MiRNA microarray data analysis

Probe summarization and normalization were calculated from raw .cel files according to manufacturer’s recommendations using miRNA QC Tool v 1.1.1.0. Data quality was also checked using Box-and-Whisker-plots that showed expected normalized means and standard deviations among the datasets, except for five probes identified as outliers, which were removed from further analysis. MiRNAs were first filtered so that analysis was performed with only well measured genes, i.e. those that contained expression in at least 2 samples. Then for unsupervised analysis (Principle Component Analysis or 2-way hierarchical clustering) three different ranking and filtering methods were used to identify a list of miRNAs that contained the most variance: 1. Coefficient of variation (CV); 2. Standard deviation; 3. Filtering for at least 2-3 samples with 1-2 standard deviations above and below the mean. After filtering, Data was log_2_ transformed, and Pearson’s correlations were iteratively calculated between all miRNA expression values for all probes and samples for 2-way hierarchical clustering. For supervised analysis, a Student’s *t*-test was used to identify differentially expressed corneal miRNAs between normal, DM, and DR patients. The False Discovery Rate or q values were calculated using the Storey method to adjust for type 1 errors with a predicted false discovery rate of < 20% [32]. The microarray data are MIAME compliant and have been deposited into the NCBI Gene Expression Omnibus. Data can be accessed through http://www.ncbi.nlm.nih.gov/geo (accession number: GSE52233).

### Quantitative Real-Time RT-PCR

Ten nanograms of total RNA was reverse transcribed (RT) using Taqman microRNA RT kit (Life Technologies). Q-PCR was carried out using Taqman 2X universal PCR master mix and 20X MicroRNA Assays (Life Technologies). Briefly, 10 ng of total RNA was used per 15 μl reaction with the RT master mix in a ratio of 5 μl RNA: 7 μl master mix and 3 μl of the specific miRNA RT primers. Real-time Q-PCR was carried out in MicroAmp Optical 96-well plate using Taqman 2X universal PCR master mix (no AmpErase UNG) along with Taqman 20X MicroRNA Assays (Life Technologies). Each well contained 1.33 μl of 1:15 diluted RT reaction, 1X Taqman PCR master mix and 1X specific primers for each miRNA, which is designed to detect and quantify mature miRNAs in real time using 7300 PCR System (Life Technologies). Each sample was run in triplicate. Signals were normalized to U6 housekeeping miRNA run simultaneously. A comparative threshold cycle (Ct) method (ΔΔCt) was used to calculate relative miRNA expression between normal and diabetic samples. 

### In situ Hybridization

Detection of corneal miR-146a was performed using a locked nucleic acid (LNA) modified probe (Exiqon, Woburn, MA) essentially as previously described [33]. Donor corneas embedded in OCT were sectioned at 8 µm onto Superfrost Plus slides (Fisher Scientific, Pittsburgh, PA), air dried at room temperature (RT) for 30 min, then fixed in 4% paraformaldehyde at RT for 10 min. Slides were washed three times in RNase free 1X PBS followed by gentle stirring in ~26 mM acetic anhydride solution for 10 min. Slides were subsequently washed once in RNase free 1X PBS and digested in 5 μg/ mL Proteinase K in diethyl pyrocarbonate (DEPC) treated water for 5 min at RT. After washing in RNase free 1X PBS three times for 3 min the slides were transferred to a hybridization chamber containing blotting paper (VWR, Radnor, PN) soaked in 50% formamide and 5X saline sodium citrate (SSC). 700 μL of hybridization buffer was added to each slide and they were incubated at RT for ~5 hours. Digoxigenin (DIG) double labeled (5’ and 3’) miR-146a LNA probe (40 nM final concentration) was heated to 80°C for 5 min in denaturing hybridization buffer, cooled on ice, and 150 µL was pipetted onto each slide. The slides were then cover slipped and allowed to hybridize overnight at 54°C in a hybridization chamber. Next, slides were soaked in 60°C 5X SSC to remove cover slips followed by incubation in 0.2X SSC at 60°C for 1 hr. Finally, slides were equilibrated in 0.1 M Tris pH 7.5/0.15 M NaCl (Buffer B1) for 10 min before blocking with 500 µL blocking solution (10% FCS in Buffer B1) per slide for 1 hr in a humidified chamber at RT. Immunohistological detection of miR-146a was performed by adding 500 μL of 1:2000 Anti-DIG Fab conjugated to alkaline phosphatase (Roche Diagnostics, Indianapolis, IN) in blocking solution to each slide and incubating at 4°C overnight. Slides were washed in Buffer B1 three times for 5 min each and then equilibrated in Buffer B3 (0.1 M Tris pH 9.5/0.1M NaCl/50 mM MgCl_2_; 0.45 μm filtered using a SFCA membrane) for 10 min at RT. Slides were placed in a humidified chamber at RT in the dark and 150 μL of a NBT/BCIP (Roche Diagnostics) alkaline phosphatase substrate solution was used to detect miRNA-146a expression. Color formation was monitored with an Olympus BX40 microscope (Olympus USA, Melville, NY), and pictures were taken with a MicroFire digital camera (Optronics, Goleta, CA) operated using PictureFrame software. Reactions were terminated by washing slides 3 times in 1X PBS plus 0.1 % Tween 20 (PBT), 10 min each at RT. Slides were mounted and cover slipped in 50% 1X PBS/50% glycerol.

### Cell Culture and Transfection

Telomerase-immortalized HCEC [34] were grown on type IV collagen-coated plates in EpiLife® serum free medium (Life Technologies) with human corneal growth supplement (HCGS) at 37°C, 5% CO_2_. Sixty percent confluent HCEC were transfected with pre-optimized concentration (30-50 nM) of human pre-miR^TM^miRNA precursors (h-miRs) or their specific inhibitors (antagomirs) using Lipofectamine 2000 (Life Technologies). Seventy-two hours after transfection the cells were used for further analysis. Total RNA was extracted for Q-PCR to assess miRNA expression changes upon transfection.

### In vitro Wound Healing Assay

Confluent transfected cells were “scratch wounded” with a P200 pipette tip [35]. Wound closure was monitored by phase contrast microscopy. At regular intervals images were taken over 24 hrs and analyzed with ImageJ software. Average wound area relative to the initial wounding (0 hr) was determined in three independent triplicate assays and was compared to control cells transfected with Cy3™-labeled random sequence pre-miRNA (negative control). 

### Western Blot Analysis

Wounded HCEC were dissolved in Laemmli’s buffer with 1% SDS, 5% 2-mercaptoethanol (Life Technologies), and proteinase inhibitors (Sigma-Aldrich, St. Louis, MO). For Western blot analysis, 8% to 16% gradient Tris-glycine SDS polyacrylamide gels were used (Life Technologies). Gel loading was normalized using monoclonal mouse antibodies AC-74 to β-actin or TUB 2.1 to β-tubulin (Sigma-Aldrich). After transfer to nitrocellulose membranes, blots were blocked in 5% defatted milk and incubated with primary antibodies, rabbit anti-p-EGFR (44-784G, Life Technologies), mouse anti-EGFR (2239, Cell Signaling Technology, Danvers, MA), mouse anti-p-p38 (ab50012, Abcam, Cambridge, MA), rabbit anti-p38 (9212, Cell Signaling) or rabbit anti-pAkt (9271, Cell Signaling). Alkaline phosphatase–conjugated (Jackson ImmunoResearch Laboratories, West Grove, PA) or horseradish peroxidase (HRP)-conjugated secondary antibodies (Santa Cruz Biotechnology, Dallas, Texas) were used with their standard substrates BCIP/NBT (Life Technologies) or Pierce ECL Western Blotting substrate (Thermo Fisher Scientific, Rockford, IL), respectively.

### Immunocytochemistry

Cultured cells were fixed in 100% methanol at –20°C for 10 min, blocked for 1 h in 2% BSA, and 0.05% Triton X-100 in PBS at room temperature in a humidity chamber. The slides were incubated with anti-p-EGFR, anti-p-p38 or anti-pAkt primary antibodies in blocking solution overnight at 4°C, followed by cross-species adsorbed secondary antibodies conjugated with fluorescein or rhodamine (EMD Millipore, Billerica, MA). For each marker the same exposure time was used when photographing stained sections. Negative controls without a primary antibody were included in each experiment.

### Statistical analysis

Experiments were analyzed using two-sided Student’s *t*-test for two groups, or ANOVA for three or more groups, with *p* < 0.05 considered significant. 

## Results

### Microarray data analysis

Out of 841 human miRNAs on the array, a total of 196 miRNA gene families (254 miRNAs) were identified as expressed in at least two or more of the samples (Table S1). For unsupervised analysis, various ranking and filtering methods were used in combination with principal component analysis and two-way hierarchical clustering to identify dominant miRNA expression signatures associated with the samples (see Methods). Each analysis produced independent sample clustering patterns with no dominant gene expression patterns, which suggests some of the gene expression differences might also be associated with high individual variability such as diabetes duration, ethnicity, age, etc. When comparing all diabetic (with or without DR) to non-diabetic samples for differential miRNA expression, we found 29 miRNAs to be up- or down-regulated 1.5 - 3.5 fold in diabetic corneas (Table 2). Though most of the expression differences were not statistically significant, five miRNAs had expression differences of more than 2-fold. Of these, miR-424 and miR-146a had from 2- to 3.5-fold higher expression in diabetic corneas. Interestingly, miR-424 was the most significantly differentially expressed microRNA between DM and normal corneas (p < 0.003, q < 0.2). MiR-146a and miR-424 were among the highest increased in expression in diabetic patients and, because of their putative roles in cell migration, warranted further investigation. 

**Table 2:** Fold changes in select miRNAs in diabetic central corneas as revealed by miRNA microarray analysis.

MicroRNA	DM/Normal, fold change	Functional roles
hsa-miR-146a	3.5	Inflammatory responses [49], hematopoiesis [50,51], migration and proliferation [42]
hsa-miR-21	2.5	OncomiR, development, and inflammation [65], vascular remodeling [66]
hsa-miR-509-3p	-2.2	*CFTR* post transcriptional regulation [67], migration, proliferation, apoptosis, tumor suppressor [68]
hsa-miR-143	-2.1	Cardiac morphogenesis [69], migration, invasion [70], differentiation [71]
hsa-miR-424	2.0	Migration & invasion [54], angiogenesis [72]
hsa-miR-297	2.0	Cancer multidrug resistance [73]
hsa-miR-92b	1.9	Differentiation, proliferation [74], invasion [75],
hsa-miR-145	-1.9	Differentiation [28,76], apoptosis, proliferation, invasion [76]
hsa-miR-346	1.8	Anti-inflammation [77]
hsa-miR-487b	-1.8	Proliferation & invasion [78], oncogenesis [79]
hsa-miR-126	-1.8	Angiogenesis [80], cell growth, invasion, migration [81]
hsa-miR-34c-5p	1.8	Tumor suppression, apoptosis [82]
hsa-miR-34b	1.8	Tumor suppression, demethylation [83]
hsa-miR-663	1.8	Proliferation & cell cycle regulation [84], tumor suppression [85]
hsa-miR-503	1.7	Proliferation & cell cycle regulation [86], apoptosis [87]
hsa-miR-181c	-1.6	Inflammatory response [88], differentiation [89]
hsa-miR-1281	1.6	Muscle-invasive bladder cancer [90]
hsa-miR-595	1.5	Tumor suppression [90]
hsa-miR-933	-1.6	Superficial spreading melanoma [91]
hsa-miR-1300	-1.6	Unknown
hsa-miR-664	-1.6	Apoptosis [92]
hsa-miR-1231	1.6	Unknown
hsa-miR-378	-1.6	Cellular growth [93], proliferation, migration, angiogenesis [94]
hsa-miR-200a	-1.6	Migration, differentiation [95-96]
hsa-miR-378	-1.6	Unknown
hsa-miR-934	-1.5	Regulation of barrier function [97]
hsa-miR-7-1	-1.5	Tumor suppression [98]
hsa-miR-149	-1.5	Proliferation, vascular, lymph node, and nerve invasion [99]
hsa-miR-181a	-1.5	Inflammatory responses [88], migration, proliferation [100]

### Validation of differentially expressed miRNAs by Q-PCR

**Figure 1:**
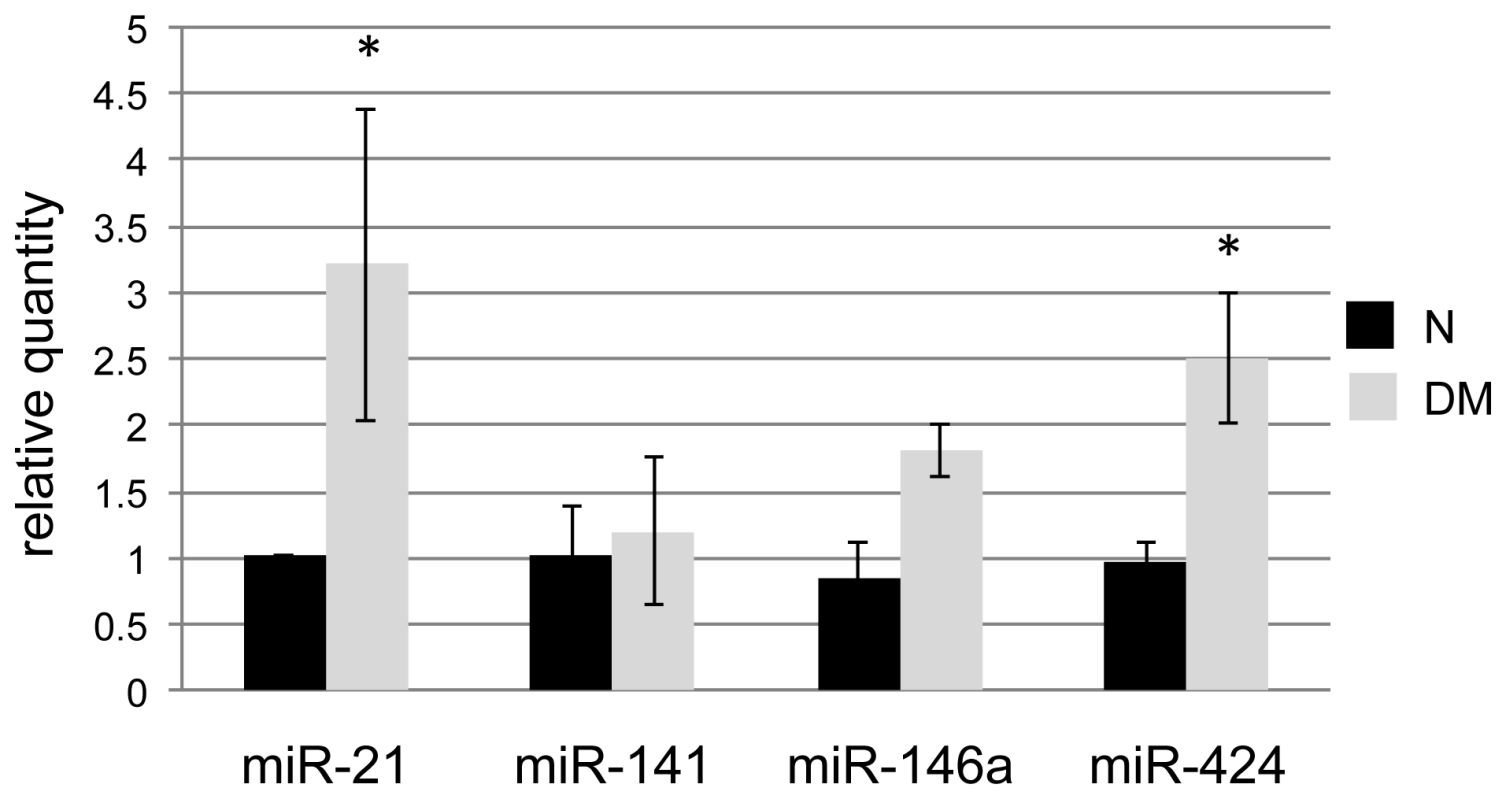
Q-PCR validation of differentially expressed miRNAs. Independent validation of differentially expressed miRNAs in *ex* *vivo* diabetic corneas by Q-PCR. N, normal; DM, diabetic. Note increased expression of three miRNAs in diabetic samples. Bars represent SEM.

We selected two miRNA (i.e. miR-146a, and miR-424) from the 254 miRNA expressed in the central human cornea that we hypothesized to have roles in disease based on the following criteria: fold change and biological inference. We also confirmed the expression of miR-21 and miR-141 with 2.5-fold and no change in diabetic cornea, respectively, according to microarray data. Results of Q-PCR analysis of expression of these candidate miRNAs are shown on Figure 1. miR-21, -146a and -424 were all found to be elevated in diabetic and DR corneas, whereas miR-141 displayed no change compared to normal corneas, in accordance with microarray data. 

### Cellular localization of miR-146a in the cornea

**Figure 2:**
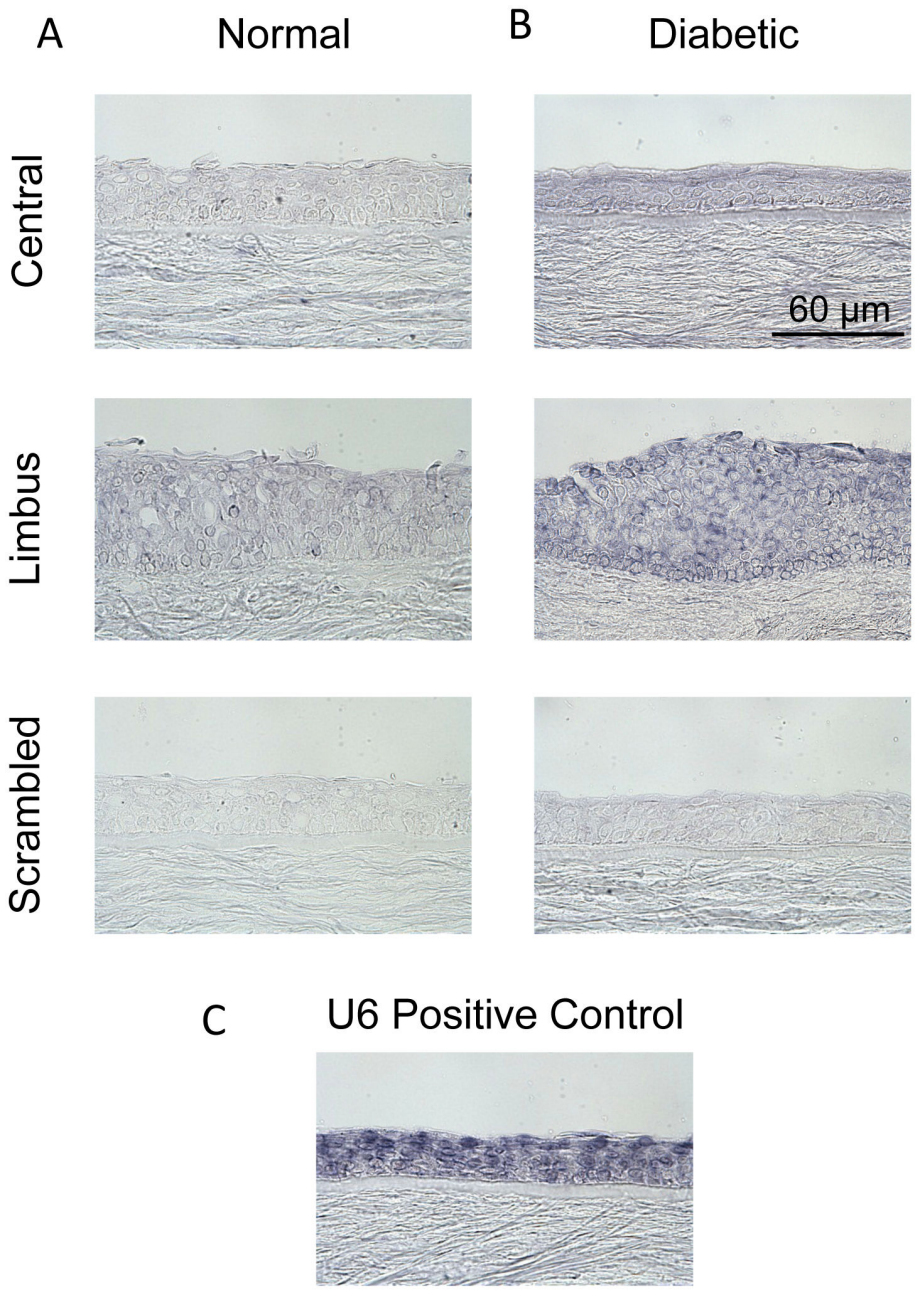
MiR-146a expression in the cornea. *In* *situ* hybridization with locked nucleic acid (LNA) modified probes was performed on sections of normal (A) and diabetic (B) corneas compared to scrambled sequences (with Cy3-labeled pre-miRNA). Diabetic corneas show somewhat elevated expression both in the central cornea and limbus. In the diabetic corneas, the miR-146a signal is stronger in the limbus than in the central cornea. Positive control in normal cornea (with U6 miRNA) is presented in panel C. Bar = 60 μm.

*In situ* hybridization was performed to determine cellular localization of miR-146a in normal and diabetic human corneas. As shown in Figure 2A, in normal human corneas miR-146a ISH yielded a diffuse and weak signal both in the central and limbal cornea. Diffuse reaction has been described for miRNA previously and is compatible with the cytoplasmic localization of the mature miRNA [36]. Diabetic corneas showed a stronger signal, especially in the limbus, where it was also more pronounced in the basal cell layer (Figure 2B). U6 miRNA was used as a positive control in normal cornea (Figure 2C).

### Assessment of differentially expressed miRNAs using HCEC wound healing assay

**Figure 3:**
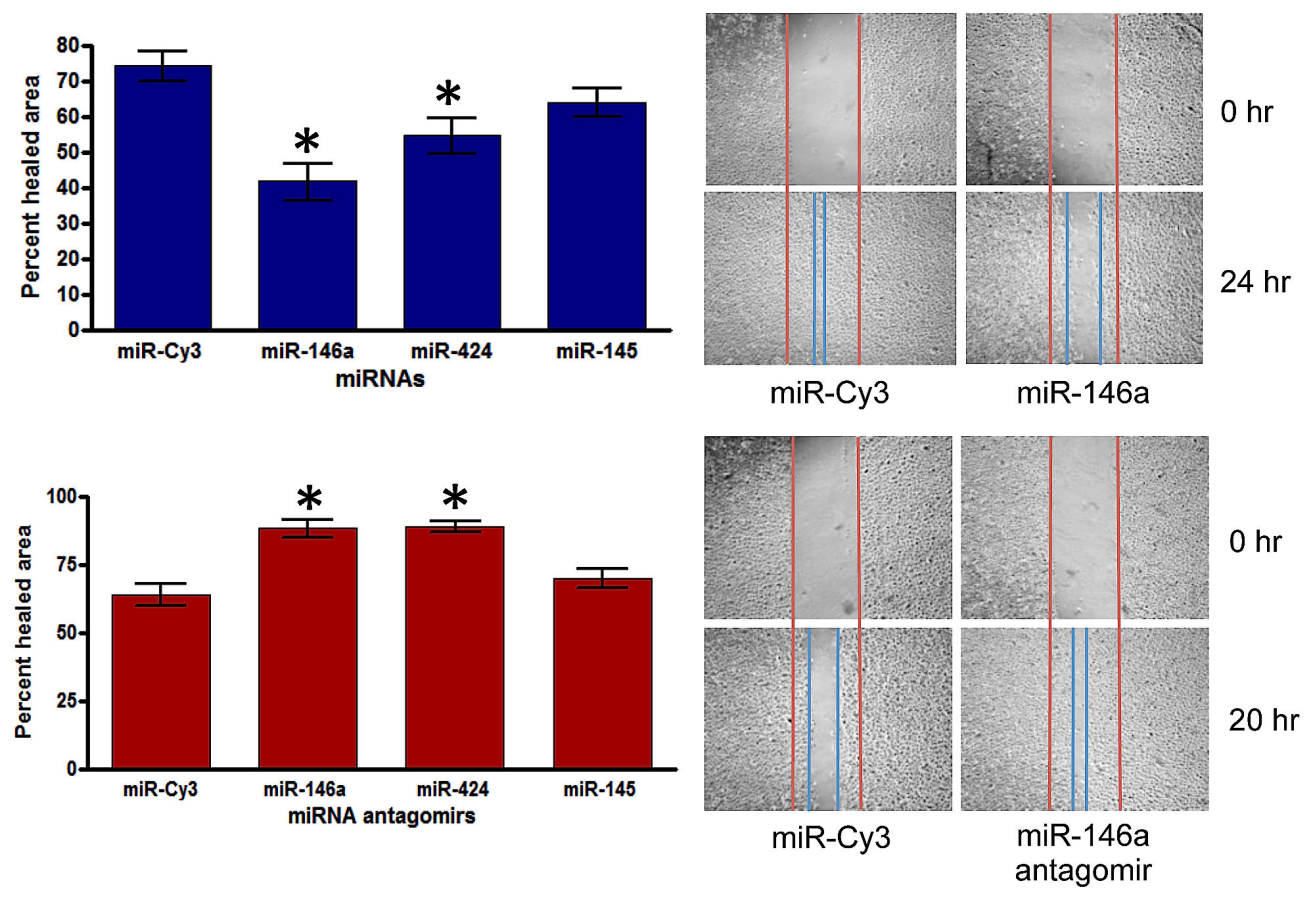
Effect of miRNAs on wound healing. A. HCEC were transfected with pre-miR-146a, -424, and -145 (top panel) or their antagomirs, pre-miRNA inhibitors (bottom panel); for control, irrelevant Cy3-labeled pre-miRNA was used. HCEC were scratch wounded as indicated in Methods. Wound closure was quantified using ImageJ software at 20 hr after wounding for pre-miRNA transfected cells and at 24 hr for pre-miRNA antagomir transfected cells. Bars represent SEM. *, p < 0.05 vs. miR-Cy3. B. miR-146a decreased wound closure, whereas its antagomir significantly increased it at 20 h compared to control. Values are expressed as percentage of the initial wound area. Data are means of three independent experiments in triplicate.

To examine the function of differentially expressed miRNAs in diabetic cornea, a telomerase-immortalized HCEC cell line was used. HCEC have similar characteristics to the primary human corneal epithelial cells with regards to cell cycle protein and cytokeratin expression profile [34]. To test whether miR-146a or miR-424 overexpression can affect the time course of wound closure, HCEC were transfected with a pre-miR-146a or pre-miR-424 precursors or, alternatively, with respective antagomirs. We verified the increased miRNA expression levels in HCEC transfected with mimic and decreased levels when using antagomirs by Q-PCR (data not shown). Confluent transfected cells were scratch wounded, and wound closure was monitored by phase contrast microscopy. In control cells transfected with irrelevant Cy3-labeled pre-miRNA, wound beds were almost covered by 24 h, whereas pre-miR-146a or pre-miR-424 significantly inhibited wound closure in HCEC (Figure 3A). To further test the specificity of miR-146a and miR-424 effects in wound healing, HCEC were transfected with their specific antagomirs. As shown in Figure 3B, antagomirs significantly enhanced wound healing by HCEC vs. control cells transfected with Cy3-labeled pre-miRNA. This suggests a physiological role of miR-146a and miR-424 in wound healing.

### MiR-146a and miR-424 activate wound healing-related signaling molecules

**Figure 4:**
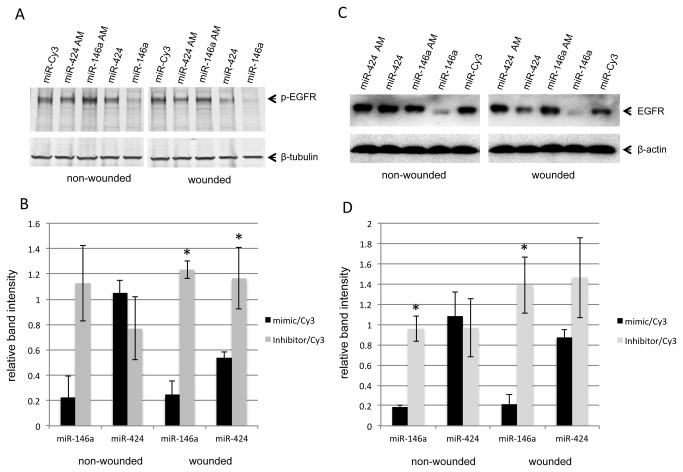
Western blot analysis of EGFR and p-EGFR expression in transfected HCEC. Total extracted protein from HCEC transfected with pre-miRNA precursors or their inhibitors (antagomirs, AM) and Cy3-labeled pre-miRNA (control) was separated on gradient SDS-PAGE gels, transferred to nitrocellulose and probed with antibodies to EGFR or p-EGFR. Antibodies to β-tubulin or β-actin were used as equal loading controls and for semi-quantitation. In both wounded and non-wounded HCEC, miR-146a mimic treatment decreased whereas its antagomir increased protein levels of p-EGFR (A,B). MiR-424 mimic and antagomir treatments did not change the expression of p-EGFR in non-wounded cells (A,B). However, in wounded cells, miR-424 mimic decreased whereas its antagomir increased protein levels of p-EGFR (A,B). In both wounded and non-wounded HCEC, miR-146a mimic treatment decreased whereas its antagomir significantly increased protein levels of total EGFR (C,D). MiR-424 mimic and antagomir treatments did not significantly change the expression of EGFR both in wounded and non-wounded cells (C,D). Band intensities were quantified using ImageJ software and plotted relative to the loading controls. Blots in A were developed with alkaline phosphatase system, blots in C, with ECL reagent. *, p < 0.05.

**Figure 5:**
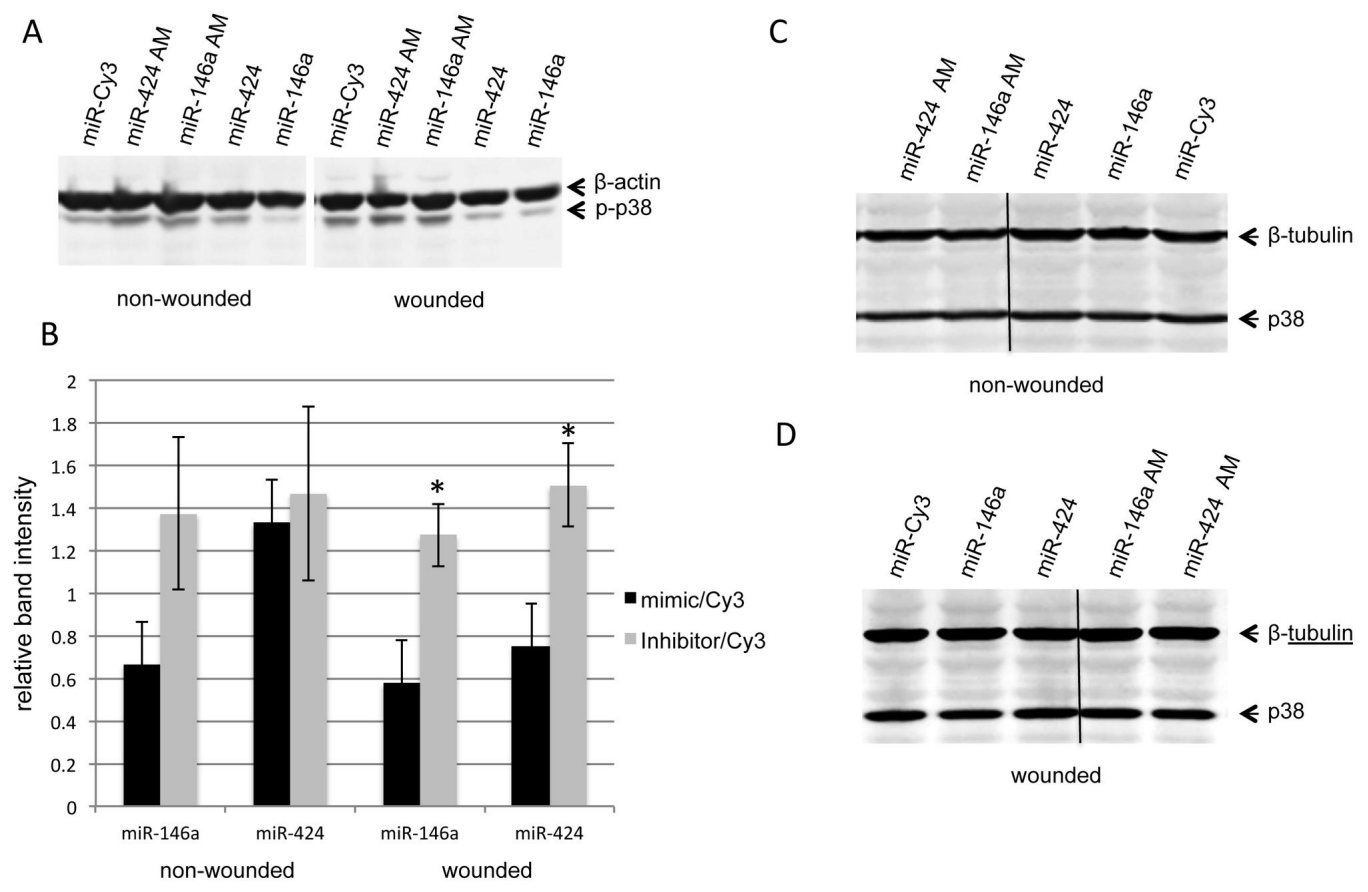
Western blot analysis of p38 and p-p38 expression in transfected HCEC. Total extracted protein from HCEC transfected with pre-miRNA precursors or their inhibitors (antagomirs, AM) and Cy3-labeled pre-miRNA (control) was separated on SDS-PAGE gels, transferred to nitrocellulose and probed with antibodies to p38 or p-p38. Antibodies to β-actin or β-tubulin were used as equal loading control and for semi-quantitation. In both wounded and non-wounded HCEC, miR-146a mimic treatment decreased whereas its antagomir increased protein levels of p-p38 (A,B). MiR-424 mimic and antagomir treatments did not change the expression of p-p38 in non-wounded cells (A,B). However, in wounded cells, miR-424 mimic decreased whereas its antagomir increased protein level of p-p38 (A,B). MiR-146a and miR-424 mimic and their antagomir treatments did not change the expression of total p38 both in non-wounded (C) and wounded cells (D). Band intensities were quantified using ImageJ software and plotted relative to the loading controls. Vertical lines in C and D separate different parts of the same gel. Blots were developed with alkaline phosphatase system. *, p < 0.05.

**Figure 6:**
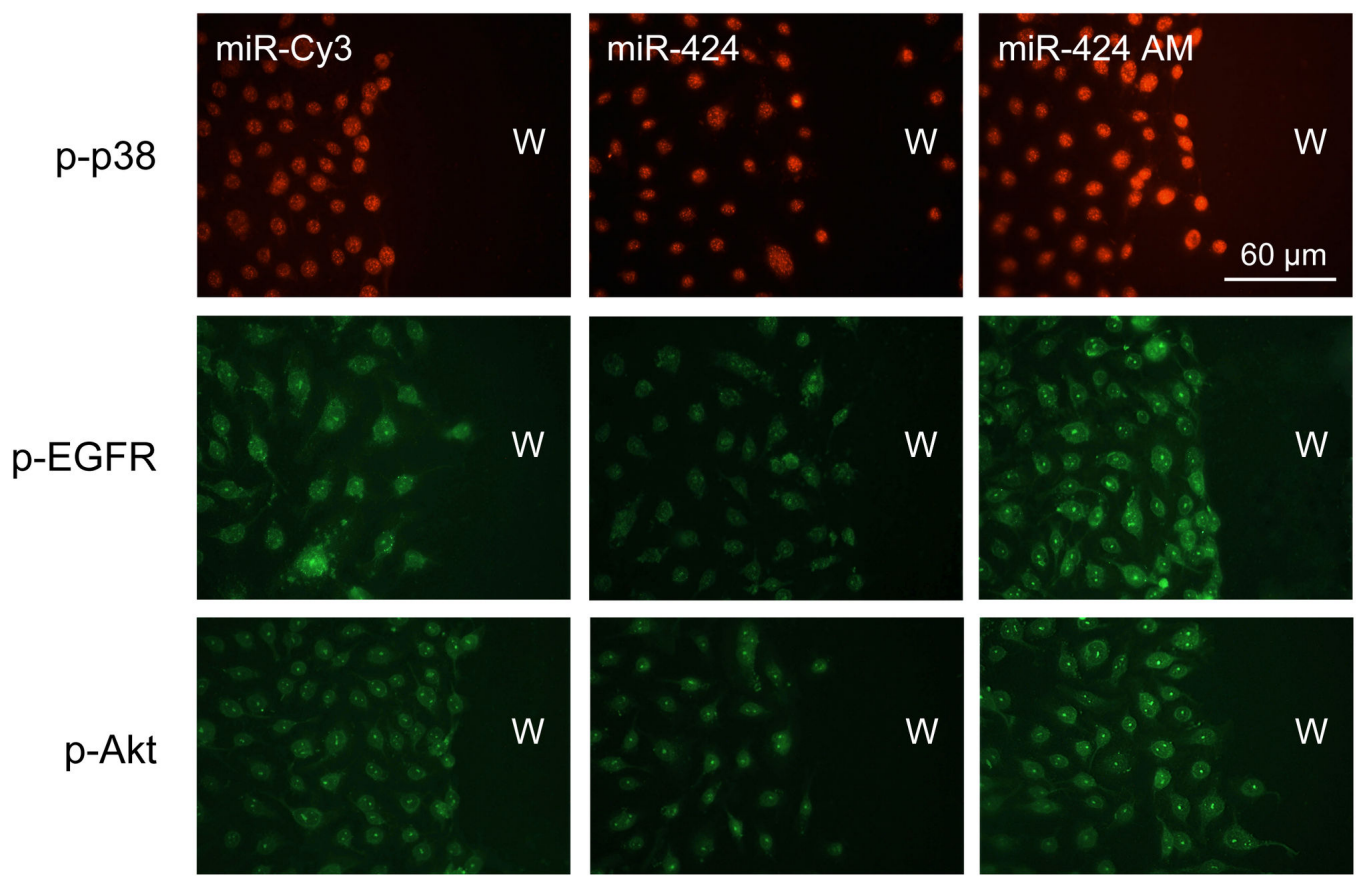
MiR-424 and wound healing-related signaling molecules. In wounded HCEC cultures, miR-424 elevated in diabetes decreased staining for p-p38, p-Akt and especially, for p-EGFR. Conversely, antagomir (AM) treatment increased the expression of all tested activated signaling intermediates above control levels. Exposure times are the same for all panels in a row. W, wound area. Bar = 60 μm.

To gain insight into the molecular mechanisms associated with miR-146a and miR-424 overexpression or their downregulation using antagomirs, we investigated how these miRNAs can influence signaling molecules previously associated with corneal epithelial wound healing [37-41]. The effect of both miR-146a and miR-424 mimics or antagomirs (AM on Figures 4-6) on the expression of EGFR, p38 MAP kinase, their phosphorylated/activated (p) forms (p-EGFR and p-p38), and p-Akt in control and wounded HCEC was examined. Confluent HCEC transfected with either miR-146a, miR-424 or their antagomirs were wounded and the expression of total and activated signaling molecules was determined by Western blot (Figures 4 A,C and 5 A,C,D) and immunostaining (Figure 6). In wounded HCEC cultures, both miR-146a and miR-424 antagomirs increased protein levels of p-EGFR (Figure 4 A,B) and p-p38 (Figure 5 A,B) and cell staining for respective proteins (Figure 6). Conversely, miR-424 and especially miR-146a mimics decreased the expression of both activated signaling intermediates below control levels (Figures 4 A,B and 5 A,B). The same trend of changes was observed for protein level of p-Akt in both miR-146a and miR-424 mimic and antagomir transfected HCEC, respectively, although it did not reach significance (data not shown here). In non-wounded HCEC, miR-146a mimic decreased the expression levels of p-EGFR (Figure 4 A,B) and p-p38 (Figure 5 A,B), whereas its antagomir increased the expression of both. However, miR-424 mimic and antagomir treatments in non-wounded cells did not change the expression of both activated signaling intermediates tested (Figures 4 A,B and 5 A,B). The expression of total EGFR and p38 in mimic- or antagomir-transduced control and wounded cultures was also examined. MiR-146a mimic or its inhibitor/antagomir changed total EGFR in both wounded and non-wounded transfected HCEC (Figure 4 C,D). The mimic transduction caused a decrease in total EGFR, but the antagomir expectedly produced the opposite effect. This result was anticipated, since EGFR is one of the targets for miR-146a [42-44]. Transduction of HCEC with miR-424 mimic or its inhibitor led to some decrease in total EGFR in wounded cultures only, although this effect did not reach statistical significance (Figure 4 C,D). In non-wounded cultures, miR-424 mimic or antagomir did not show any effect (Figure 4 C,D). Total p38 MAPK did not show any changes in wounded or non-wounded HCEC transfected with either miR-146a or miR-424 mimics and their inhibitors (Figure 5 C,D). Overall, the data suggest that miR-424 is more related to the regulation of wound healing per se, whereas miR-146a can alter p38 and EGFR signaling even without an active healing process.

## Discussion

Diabetes as a systemic disease significantly affects all eye structures including the cornea. Epithelial basement membrane, epithelial-stromal interactions and epithelial cell functions such as wound healing, and expression of limbal stem cell markers are impaired in corneas of diabetic patients [38-40]. To better understand the mechanisms responsible for these abnormalities, we have attempted to identify the alterations in microRNA expression in DM and DR human corneas in comparison to normal corneas using Affymetrix® GeneChip® miRNA microarrays. In this study, we asked whether these microRNAs were relevant to the diabetic state of the cornea and what could be the mechanisms of their engagement. Recently, regulatory roles of miRNAs in normal and diseased eyes have been demonstrated [26,28,29,45]. However, most of the reports focused on the analysis of miRNAs in the retina and considerably less is known about the cornea.

In general, by interrogating the entire miRNA expression profile in diabetic and healthy corneas, we identified many corneal miRNAs that were not shown previously as expressed in the cornea. Some if not all of these may have roles in corneal development, function, or disease. Notably, we observed very small global and even quantitative changes in miRNA expression profiles between normal and diabetic central corneas, which corroborates our recent preliminary result of miRNA profiling in diabetic and normal central and limbal human corneas using deep sequencing [46]. We believe that instead of massive changes in miRNA profiles, there are subtle affects in specific miRNAs. These small alterations, however, may contribute to the disease progression. It is possible that a few dysregulated miRNAs acting on several targets each could have cascading effects dysregulating the expression of genes and significantly affecting cellular function. To test this hypothesis, we identified and functionally characterized some dysregulated miRNA candidates in diabetic corneas. We confirmed that three selected miRNAs showed consistently increased expression in human diabetic central corneas by a more sensitive and precise assay such as Q-PCR using independent samples for validation. 

The contribution of some miRNAs to the regulation of wound healing, which is impaired in diabetic corneal epithelium, has been recently described. MiR-483-3p was shown to control skin keratinocyte growth arrest at the final steps of reepithelialization [47], miR-210 upregulation by hypoxia decreased keratinocyte proliferation and impaired the wound closure [48], and miR-205 affected corneal keratinocyte migration by downregulating lipid phosphatase SHIP2, which contributes to epithelial wound healing [27]. Our study reveals for the first time the involvement of miR-146a and miR-424 in corneal epithelial wound closure and suggests their role in delaying wound healing in diabetic cornea.

MiR-146a is an important regulator in many biological processes. It plays a major role as a modulator of the innate immune and inflammatory responses, and the pathogenesis of several autoimmune diseases [49]. It is important for the control of normal hematopoiesis [50,51], and there is growing evidence indicating that it may act as tumor suppressor for many cancers [52,53]. It has been also shown that miR-146a exerts inhibitory effects on cell migration and invasion and causes downregulation of its target EGFR and of other signaling molecules such as IRAK1 and NF-κB [42-44]. MiR-424 regulatory action as a tumor suppressor to inhibit cell migration and invasion has also been described [54]. Although the inflammatory process and hyperplasia are not major players in corneal homeostasis, the epithelial cell migration is an essential component of the wound healing process. These published observations are in agreement with our results suggesting that miR-146a may be a negative regulator of corneal epithelial wound healing. The molecular mechanisms through which miR-146a contributes to tumor development are unclear but they seem to be related to the ability of this miRNA to target some mRNAs, such as *TRAF6, IRAK1, CXCR4, NF-κB* and *EGFR* [42-44,55-57]. Direct interaction between miR-146a and its predicted target *Smad4* mRNA was also shown by luciferase assay suggesting its involvement in cell proliferation through TGF-β1/Smad signal transduction pathway [58].

Previously, we have shown that overexpression of hepatocyte growth factor (HGF) receptor *c-met* in diabetic corneas restored nearly normal epithelial wound healing times through activation of p38 MAP kinase [39]. We have further observed delayed wound healing in normal corneas upon overexpression of diabetes-upregulated proteinases that was accompanied by reduced expression of activated EGFR and Akt [38]. 

It was thus of considerable interest to elucidate the effects of miRNAs that suppress wound healing, e.g. miR-146a and miR-424, on the above-mentioned signaling molecules. Previous work has shown decreased expression of p-EGFR, p-Akt, and p-p38 in diabetic corneas; however, their total protein content did not differ significantly [37,59,60]. Since microRNA can affect target mRNA levels, we examined levels of both total and phosphorylated/activated EGFR and p38 using Western blot analysis. 

Our data indicate that both EGFR and p-38 were activated during corneal epithelial wound healing in cells transfected with both miR-424 and especially miR-146a inhibitors/antagomirs. As total EGFR expression was also reduced in both wounded and non-wounded HCEC transfected with miR-146a mimic and increased by its inhibitor/antagomir, we concluded that miR-146a negatively regulated the expression and activation of its target EGFR in HCEC but was not directly involved in wound healing process. At the same time, miR-424 mimic decreased and its inhibitor increased the levels of phosphorylated EGFR in wounded cells only. However, total EGFR did not show significant alterations in wounded or non-wounded HCEC transfected with miR-424 mimic or its inhibitor, although a tendency for a change similar to p-EGFR was seen in wounded cells. It may be suggested that miR-424 regulates wound healing in HCEC by impacting the activity of an effector upstream from EGFR. This possibility is under investigation focusing on miR-424 target Chk1, which acts as a positive regulator of EGF signaling [61]. Total p38 MAPK did not show any changes both in wounded and non-wounded HCEC transfected with either miR-146a or miR-424 mimics and their inhibitors. These findings also show that the effect of both miR-146a and miR-424 may not be attributable to their direct regulation of p38 but rather to an indirect influence on some factor upstream of p38 MAPK. This concerned the activation of major components of the corneal epithelial wound healing process, because inhibition of the EGFR or p38 signaling pathways significantly slowed epithelial migration rates [39,62]. Altered expression of EGFR-PI3K-Akt pathway and ERK has been observed in diabetic rat corneas resulting in increased apoptosis, decreased cell proliferation, and delayed corneal wound closure [37]. 

Our data are in agreement with several studies suggesting that miR-146a regulates target genes, such as EGFR, NF-κB/IKKβ or SMAD, that are involved in cell proliferation and migration during corneal wound healing [63]. Decreased expression of p-p38 in miR-146a-transfected HCEC may be due to inhibitory effect of miR-146a on NF-κB/IKKβ, which is required for cytokine-induced cell migration and wound healing through formation of an IKKβ-p38 protein complex in corneal epithelial cells [63]. Activation of Akt in corneal wound healing may be attributable to the action of the EGF/EGFR system. As EGFR was shown to modulate HGF/c-met activity, and HGF/c-met system can transactivate EGFR [64], their signaling pathways involving p-38 and PI3K-Akt may be activated concurrently leading to enhanced wound healing.

In the cornea, where Akt mediated EGFR-dependent epithelial wound healing, a similar reduction of p-Akt was observed after high glucose treatment mimicking diabetic conditions [60]. It may be suggested that somewhat decreased Akt activation upon overexpression of miR-424 could lead to decreased cell migration (retarded wound healing) because Akt is downstream of several motogenic growth factors, especially EGF [60]. Interestingly, staining for p-EGFR also decreased upon miR-424 overexpression. These results suggest that miR-424 overexpression in HCEC may interfere with EGFR signaling thereby affecting wound healing-related activation of Akt.

In summary, we used microarrays to identify candidate dysregulated miRNAs associated with diabetic corneas. These miRNAs were validated by Q-PCR and ISH in independent samples confirming that miRNA changes are associated with diabetic state of the cornea. In addition, select miRNAs, miR-146a and miR-424, increased in diabetic corneas were shown to retard directly or indirectly corneal epithelial cell wound healing in culture suggesting a functional role in disease development. These data attest to the significant role of miRNAs in regulating corneal cell behavior, and a potentially high impact of manipulating their levels for alleviation of disease symptoms. However, we are aware that a large number of samples in this cohort will be required to fully resolve the significance of a greater number of miRNAs involved in this process and that individual variations play a role in the data significance. To address these issues, performing small RNA quantitative deep sequencing with more samples may help identify other diabetes-altered miRNAs that may not be represented on the microarray.

## Supporting Information

Table S1 **List of expressed miRNAs in human cornea using microarray analysis.** A total of 196 miRNA gene families (254 miRNAs) were identified as expressed in at least two or more of the central corneas.
(DOCX)
