# Supplementary material for: Differentially Expressed Wound Healing-Related microRNAs in the Human Diabetic Cornea
Source: PLoS One. 2013 Dec 20;8(12):e84425. doi: 10.1371/journal.pone.0084425 (PMC3869828; doi:10.1371/journal.pone.0084425)
Supplement: Table S1 — List of expressed miRNAs in human cornea using microarray analysis. A total of 196 miRNA gene families (254 miRNAs) were identified as expressed in at least two or more of the central corneas. (DOCX) [file pone.0084425.s001.docx]

Table S1. List of expressed miRNAs in human cornea using microarray analysis

| MiRNA |
| --- |
| \| hsa-let-7b \| \| --- \| \| hsa-let-7d \| \| hsa-let-7g \| \| hsa-let-7i \| \| hsa-mir-100 \| \| hsa-mir-101-1 // hsa-mir-101-2 \| \| hsa-mir-103-2 // hsa-mir-103-1 \| \| hsa-mir-106a \| \| hsa-mir-106b \| \| hsa-mir-107 \| \| hsa-mir-10a \| \| hsa-mir-1180 \| \| hsa-mir-1184 // hsa-mir-1184 // hsa-mir-1184 \| \| hsa-mir-1201 \| \| hsa-mir-1202 \| \| hsa-mir-1207 \| \| hsa-mir-1224 \| \| hsa-mir-1225 \| \| hsa-mir-1228 \| \| hsa-mir-1231 \| \| hsa-mir-124-3 // hsa-mir-124-2 // hsa-mir-124-1 \| \| hsa-mir-1246 \| \| hsa-mir-125a \| \| hsa-mir-125b-1 // hsa-mir-125b-2 \| \| hsa-mir-126 \| \| hsa-mir-1260 \| \| hsa-mir-1262 \| \| hsa-mir-1263 \| \| hsa-mir-1267 \| \| hsa-mir-1268 \| \| hsa-mir-127 \| \| hsa-mir-1270 \| \| hsa-mir-1271 \| \| hsa-mir-1272 \| \| hsa-mir-1274b \| \| hsa-mir-1275 \| \| hsa-mir-128-1 // hsa-mir-128-2 \| \| hsa-mir-1280 \| \| hsa-mir-1281 \| \| hsa-mir-1285-2 // hsa-mir-1285-1 \| \| hsa-mir-1287 \| \| hsa-mir-1288 \| \| hsa-mir-129-2 // hsa-mir-129-1 \| \| hsa-mir-1290 \| \| hsa-mir-1291 \| \| hsa-mir-1299 \| \| hsa-mir-1300 \| \| hsa-mir-1301 \| \| hsa-mir-1303 \| \| hsa-mir-1307 \| \| hsa-mir-1308 \| \| hsa-mir-130a \| \| hsa-mir-130b \| \| hsa-mir-132 \| \| hsa-mir-1323 \| \| hsa-mir-134 \| \| hsa-mir-135a-2 // hsa-mir-135a-1 \| \| hsa-mir-135b \| \| hsa-mir-138-2 // hsa-mir-138-1 \| \| hsa-mir-139 \| \| hsa-mir-140 \| \| hsa-mir-141 \| \| hsa-mir-143 \| \| hsa-mir-144 \| \| hsa-mir-145 \| \| hsa-mir-146a \| \| hsa-mir-146b \| \| hsa-mir-148a \| \| hsa-mir-148b \| \| hsa-mir-149 \| \| hsa-mir-150 \| \| hsa-mir-151 \| \| hsa-mir-152 \| \| hsa-mir-155 \| \| hsa-mir-15a \| \| hsa-mir-15b \| \| hsa-mir-16-1 // hsa-mir-16-2 \| \| hsa-mir-17 \| \| hsa-mir-181a-1 // hsa-mir-181a-2 \| \| hsa-mir-181b-1 // hsa-mir-181b-2 \| \| hsa-mir-181c \| \| hsa-mir-181d \| \| hsa-mir-182 \| \| hsa-mir-1825 \| \| hsa-mir-1826 \| \| hsa-mir-183 \| \| hsa-mir-184 \| \| hsa-mir-185 \| \| hsa-mir-188 \| \| hsa-mir-18a \| \| hsa-mir-18b \| \| hsa-mir-191 \| \| hsa-mir-192 \| \| hsa-mir-193a \| \| hsa-mir-193b \| \| hsa-mir-194-2 // hsa-mir-194-1 \| \| hsa-mir-195 \| \| hsa-mir-196a-2 // hsa-mir-196a-1 \| \| hsa-mir-197 \| \| hsa-mir-198 \| \| hsa-mir-199a-1 // hsa-mir-199a-2 \| \| hsa-mir-199b \| \| hsa-mir-19a \| \| hsa-mir-19b-1 // hsa-mir-19b-2 \| \| hsa-mir-200a \| \| hsa-mir-200b \| \| hsa-mir-200b \| \| hsa-mir-200c \| \| hsa-mir-203 \| \| hsa-mir-204 \| \| hsa-mir-205 \| \| hsa-mir-20a \| \| hsa-mir-20b \| \| hsa-mir-21 \| \| hsa-mir-210 \| \| hsa-mir-212 \| \| hsa-mir-214 \| \| hsa-mir-22 \| \| hsa-mir-220c \| \| hsa-mir-221 \| \| hsa-mir-221 \| \| hsa-mir-222 \| \| hsa-mir-223 \| \| hsa-mir-23a \| \| hsa-mir-23b \| \| hsa-mir-24-2 // hsa-mir-24-1 \| \| hsa-mir-25 \| \| hsa-mir-26a-2 // hsa-mir-26a-1 \| \| hsa-mir-26b \| \| hsa-mir-27a \| \| hsa-mir-27b \| \| hsa-mir-28 \| \| hsa-mir-296 \| \| hsa-mir-297 \| \| hsa-mir-29a \| \| hsa-mir-29b-2 // hsa-mir-29b-1 \| \| hsa-mir-29c \| \| hsa-mir-301a \| \| hsa-mir-30a \| \| hsa-mir-30b \| \| hsa-mir-30c-1 // hsa-mir-30c-2 \| \| hsa-mir-30d \| \| hsa-mir-30e \| \| hsa-mir-31 \| \| hsa-mir-32 \| \| hsa-mir-320a \| \| hsa-mir-320d-1 // hsa-mir-320d-2 \| \| hsa-mir-324 \| \| hsa-mir-328 \| \| hsa-mir-330 \| \| hsa-mir-331 \| \| hsa-mir-335 \| \| hsa-mir-338 \| \| hsa-mir-339 \| \| hsa-mir-342 \| \| hsa-mir-345 \| \| hsa-mir-346 \| \| hsa-mir-34a \| \| hsa-mir-34b \| \| hsa-mir-34c \| \| hsa-mir-361 \| \| hsa-mir-362 \| \| hsa-mir-372 \| \| hsa-mir-374b \| \| hsa-mir-375 \| \| hsa-mir-376a-2 // hsa-mir-376a-1 \| \| hsa-mir-376c \| \| hsa-mir-378 \| \| hsa-mir-379 \| \| hsa-mir-382 \| \| hsa-mir-409 \| \| hsa-mir-421 \| \| hsa-mir-422a \| \| hsa-mir-423 \| \| hsa-mir-424 \| \| hsa-mir-425 \| \| hsa-mir-429 \| \| hsa-mir-432 \| \| hsa-mir-451 \| \| hsa-mir-454 \| \| hsa-mir-455 \| \| hsa-mir-483 \| \| hsa-mir-486 \| \| hsa-mir-487b \| \| hsa-mir-488 \| \| hsa-mir-489 \| \| hsa-mir-491 \| \| hsa-mir-494 \| \| hsa-mir-497 \| \| hsa-mir-500 \| \| hsa-mir-501 \| \| hsa-mir-502 \| \| hsa-mir-503 \| \| hsa-mir-504 \| \| hsa-mir-505 \| \| hsa-mir-508 \| \| hsa-mir-509-2 // hsa-mir-509-3 // hsa-mir-509-1 \| \| hsa-mir-509-3 \| \| hsa-mir-510 \| \| hsa-mir-532 \| \| hsa-mir-548a-2 // hsa-mir-548a-1 // hsa-mir-548a-3 \| \| hsa-mir-548p \| \| hsa-mir-550-1 // hsa-mir-550-2 \| \| hsa-mir-551b \| \| hsa-mir-559 \| \| hsa-mir-570 \| \| hsa-mir-574 \| \| hsa-mir-584 \| \| hsa-mir-589 \| \| hsa-mir-593 \| \| hsa-mir-595 \| \| hsa-mir-603 \| \| hsa-mir-606 \| \| hsa-mir-615 \| \| hsa-mir-625 \| \| hsa-mir-626 \| \| hsa-mir-627 \| \| hsa-mir-628 \| \| hsa-mir-629 \| \| hsa-mir-635 \| \| hsa-mir-638 \| \| hsa-mir-652 \| \| hsa-mir-658 \| \| hsa-mir-660 \| \| hsa-mir-663 \| \| hsa-mir-664 \| \| hsa-mir-665 \| \| hsa-mir-671 \| \| hsa-mir-675 \| \| hsa-mir-708 \| \| hsa-mir-720 \| \| hsa-mir-744 \| \| hsa-mir-768 \| \| hsa-mir-769 \| \| hsa-mir-874 \| \| hsa-mir-877 \| \| hsa-mir-885 \| \| hsa-mir-886 \| \| hsa-mir-921 \| \| hsa-mir-923 \| \| hsa-mir-92a-1 // hsa-mir-92a-2 \| \| hsa-mir-92b \| \| hsa-mir-93 \| \| hsa-mir-933 \| \| hsa-mir-934 \| \| hsa-mir-935 \| \| hsa-mir-936 \| \| hsa-mir-938 \| \| hsa-mir-939 \| \| hsa-mir-943 \| \| hsa-mir-96 \| \| hsa-mir-98 \| \| hsa-mir-99a \| \| hsa-mir-99b \| |
